# A Rare Twist in the Tale: Midgut Volvulus From Intestinal Non-rotation in an Adult

**DOI:** 10.7759/cureus.87999

**Published:** 2025-07-15

**Authors:** Ram Kumar, Alexander Mecheri Antony, Ganesh Guru, V Ramlakshmi, Gowthaam Ramesh

**Affiliations:** 1 General Surgery, Bharath Institute of Higher Education and Research, Chennai, IND; 2 Surgery, Sree Balaji Medical College and Hospital, Chennai, IND; 3 General Surgery, Sree Balaji Medical College and Hospital, Chennai, IND

**Keywords:** acute midgut volvulus, adult intestinal malrotation, surgical acute abdomen, whirlpool sign, emergency laparotomy

## Abstract

Intestinal malrotation is an inherited condition where the midgut is not properly rotated, and it tends to be noticed in infancy. It becomes evident in later life with unusual abdominal pain and vomiting, which happen due to occasional blockages. Consequently, there is a risk of midgut volvulus and damage to the blood supply of the bowel. For diagnosis, the best method is imaging, especially using a contrast-enhanced upper gastrointestinal series that reveals characteristic signs such as a corkscrew shape and a misplaced duodenojejunal junction. Using ultrasonography and computed tomography (CT) scans, visualization of an unusual relationship between the superior mesenteric vessel and whirlpool sign, as well as abnormal location of the jejunal artery, can be confirmed. Most surgeons use the elective Ladd's procedure for the surgery. When bowel ischemia is found, surgery must be done, and this may result in the difficulties of short bowel syndrome. Given the variable and often subtle clinical presentation, a high index of suspicion for intestinal malrotation is crucial among patients presenting with acute abdomen and intestinal ischemia, necessitating prompt surgical intervention guided by accurate clinical and radiological evaluation to improve patient outcomes.

## Introduction

Intestinal malrotation represents a spectrum of congenital conditions resulting from the failure of the midgut to complete its physiological rotation and subsequent fixation within the abdominal cavity. The absence or partial completion of a 270-degree counterclockwise rotation around the superior mesenteric vessels leads to an abnormal spatial relationship between the intestinal segments [1]. In patients with intestinal malrotation, the typical anatomical landmark of the ligament of Treitz is absent due to the aberrant positioning of the bowel. Consequently, the distal duodenum and jejunum are characteristically situated on the right side of the vertebral column, deviating from their normal left-sided location [2]. The pathophysiology of intestinal malrotation, characterized by an incomplete midgut rotation and a resultant narrow mesenteric attachment, predisposes individuals to midgut volvulus. This twisting of the bowel can lead to vascular compromise, intestinal obstruction, and ultimately, tissue necrosis. Although the clinical presentation is most common in the neonatal period, with most cases diagnosed within the first month of life, a smaller subset of patients (0.2%-0.5%) are identified in adulthood [3]. The occurrence of intestinal malrotation does not exhibit a significant gender predilection, with similar rates observed in both male and female populations [4]. In adults, intestinal malrotation commonly presents with a clinical picture of intermittent, crampy abdominal pain accompanied by bile-containing vomitus. It represents the predominant predisposing factor for midgut volvulus in this age group, and subsequent obstructive complications are often localized to the colon [5].

## Case presentation

**Figure 1:**
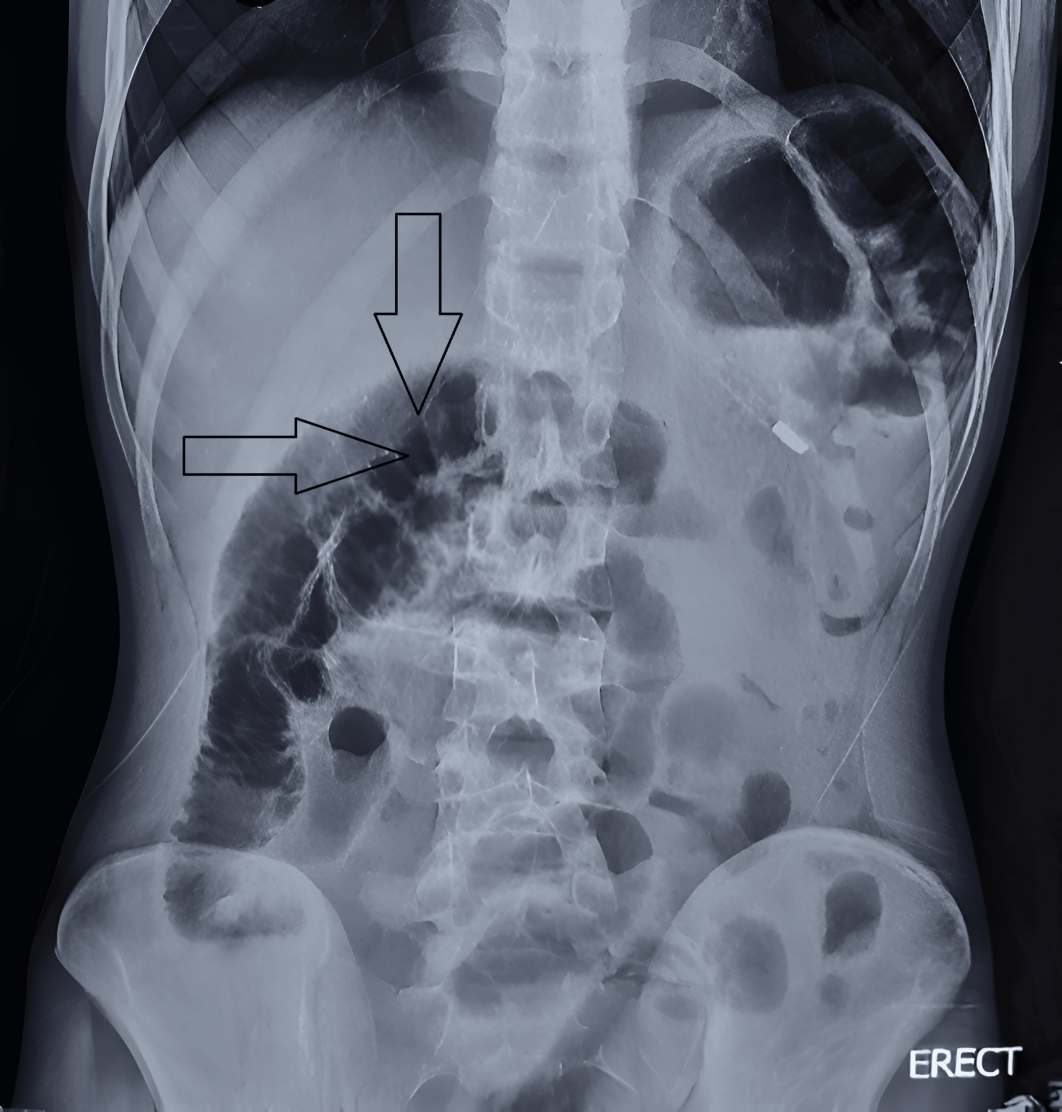
Erect abdominal X-ray showing multiple dilated bowel loops Arrows indicate multiple dilated bowel loops.

An 18-year-old male patient presented to the emergency department with a four-day history of acute, insidious onset, colicky lower abdominal pain radiating to the back, related to three episodes of non-projectile, non-bilious, non-blood-stained vomiting comprising food particles. He reported intermittent abdominal pain for the past year and a history of low-grade fever relieved by medication two days prior, along with one episode of loose stools and loss of appetite for four days. His past surgical history was observed to be significant for circumcision. Family history revealed a maternal history of a left ectopic kidney. On examination, the patient was oriented, conscious, and afebrile. Abdominal examination indicated diffuse tenderness, distension, guarding, and rigidity. Bowel sounds were exaggerated in the right lower quadrant. Digital rectal examination was unremarkable. X-ray of the abdomen showed multiple dilated bowel loops (Figure 1).

**Figure 2:**
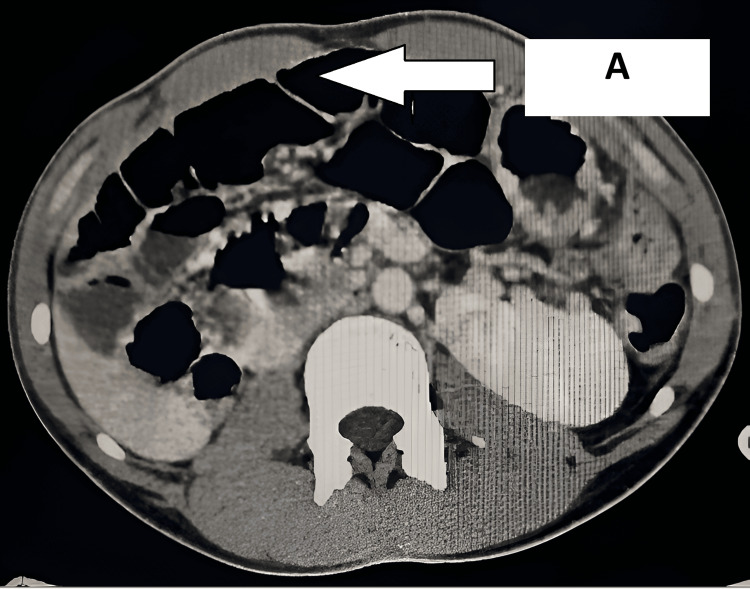
CECT of the abdomen (axial view) A: CECT of the abdomen showing a dilated bowel loop. CECT: contrast-enhanced computed tomography

**Figure 3:**
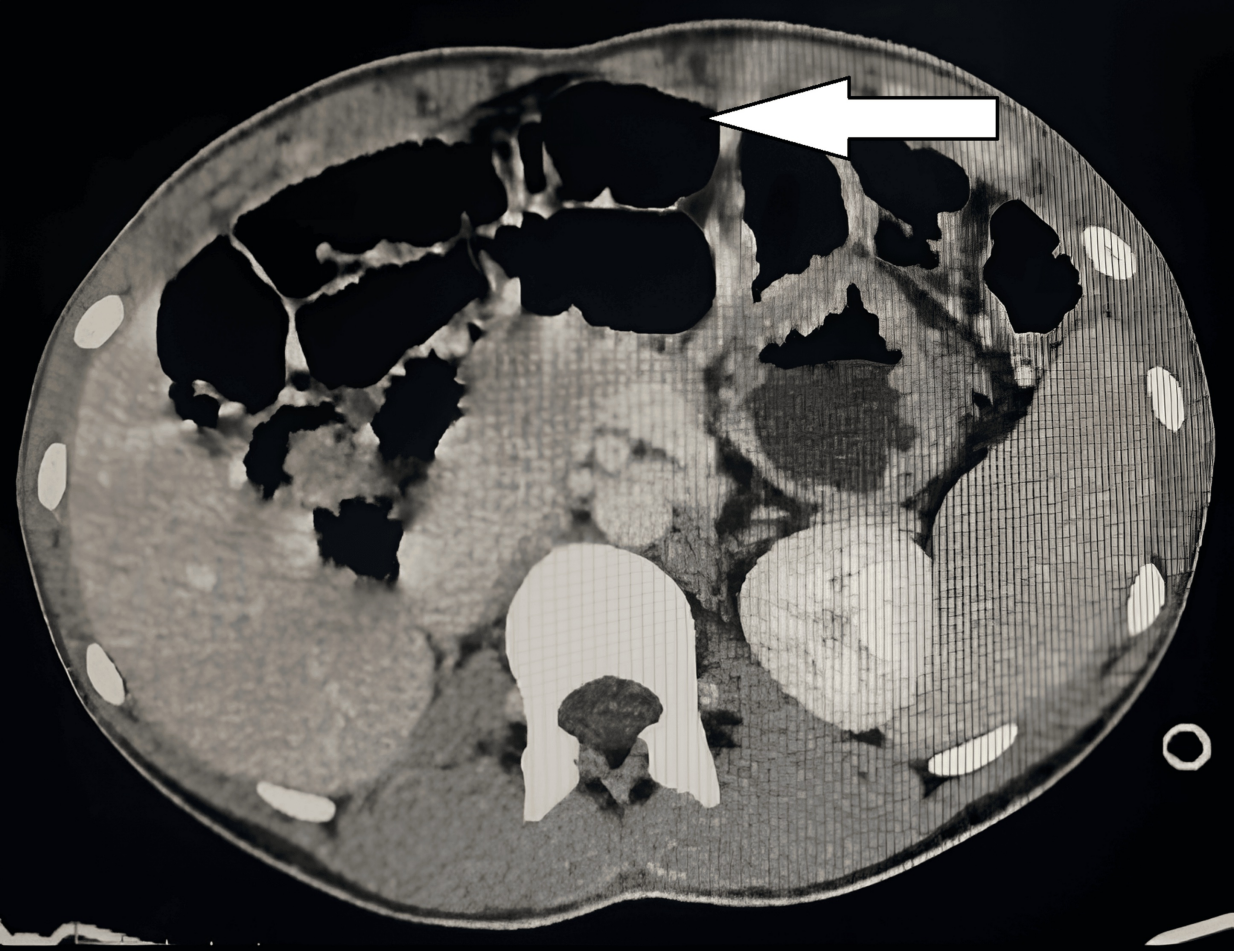
CECT of the abdomen (axial view) The arrow indicates the dilated bowel loop. CECT: contrast-enhanced computed tomography

Contrast-enhanced computed tomography (CECT) of the abdomen demonstrated the cecum and ileocecal junction in the pelvis, reversed superior mesenteric artery (SMA) and vein axis with twisting of mesenteric vessels, and small bowel loops in the right lumbar region ("whirlpool sign"), absent retromesenteric course of the D3 segment of the duodenum, and a duodenojejunal flexure on the right side. It also confirmed a right ectopic kidney cross-fused with the left kidney (Figures 2, 3).

**Figure 4:**
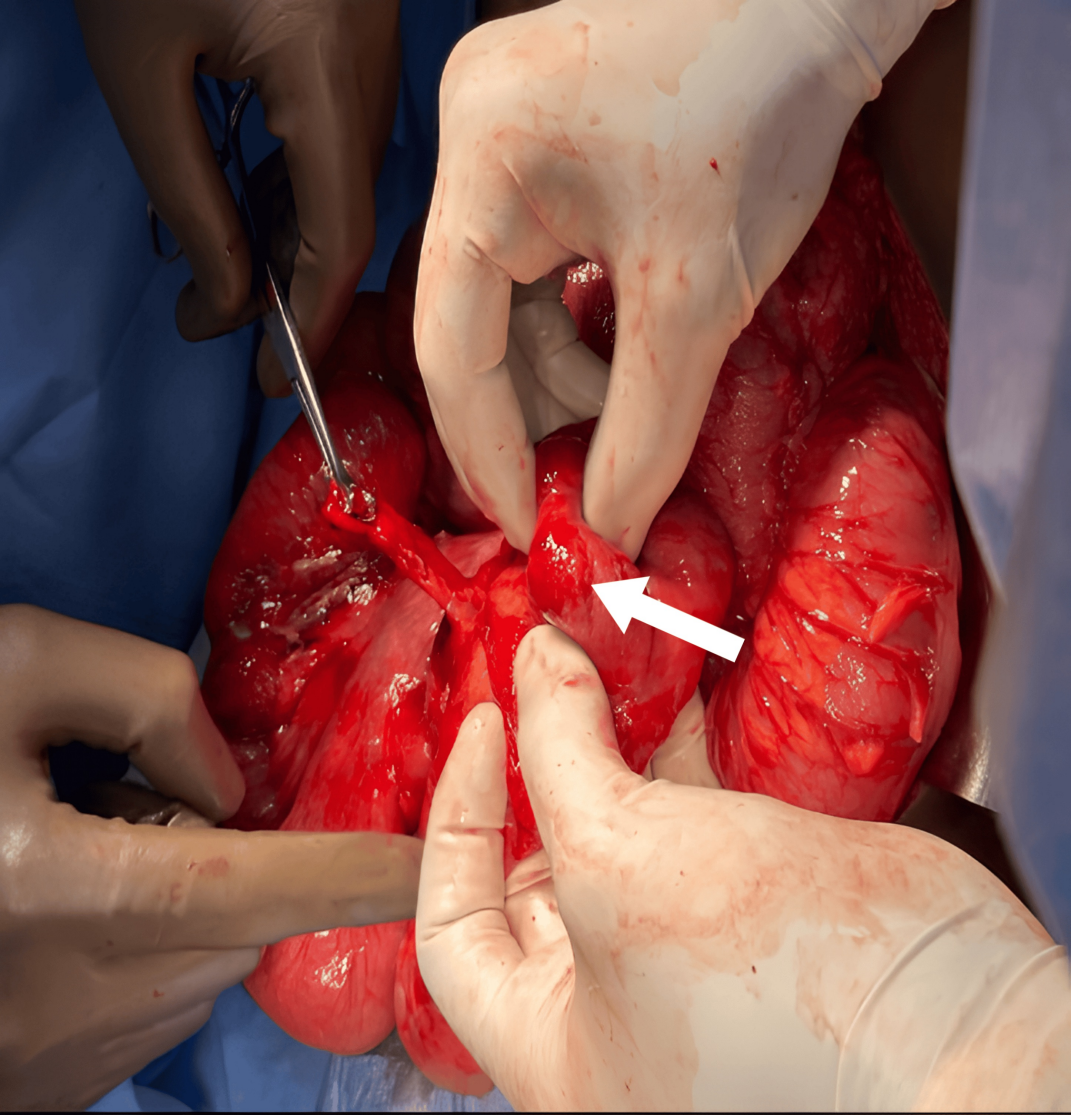
Intraoperative image The arrow points to the cecum.

**Figure 5:**
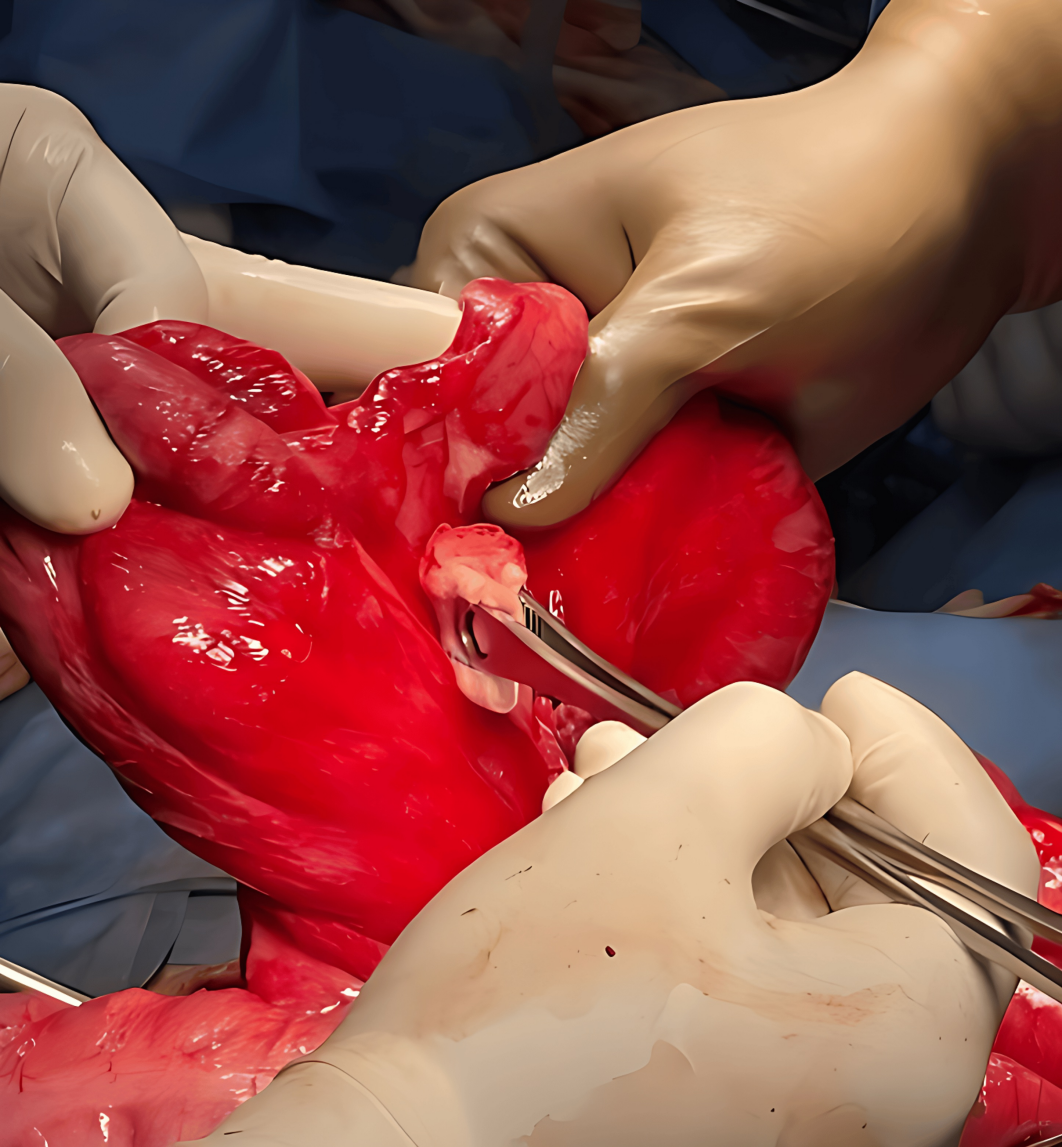
Intraoperative image

**Figure 6:**
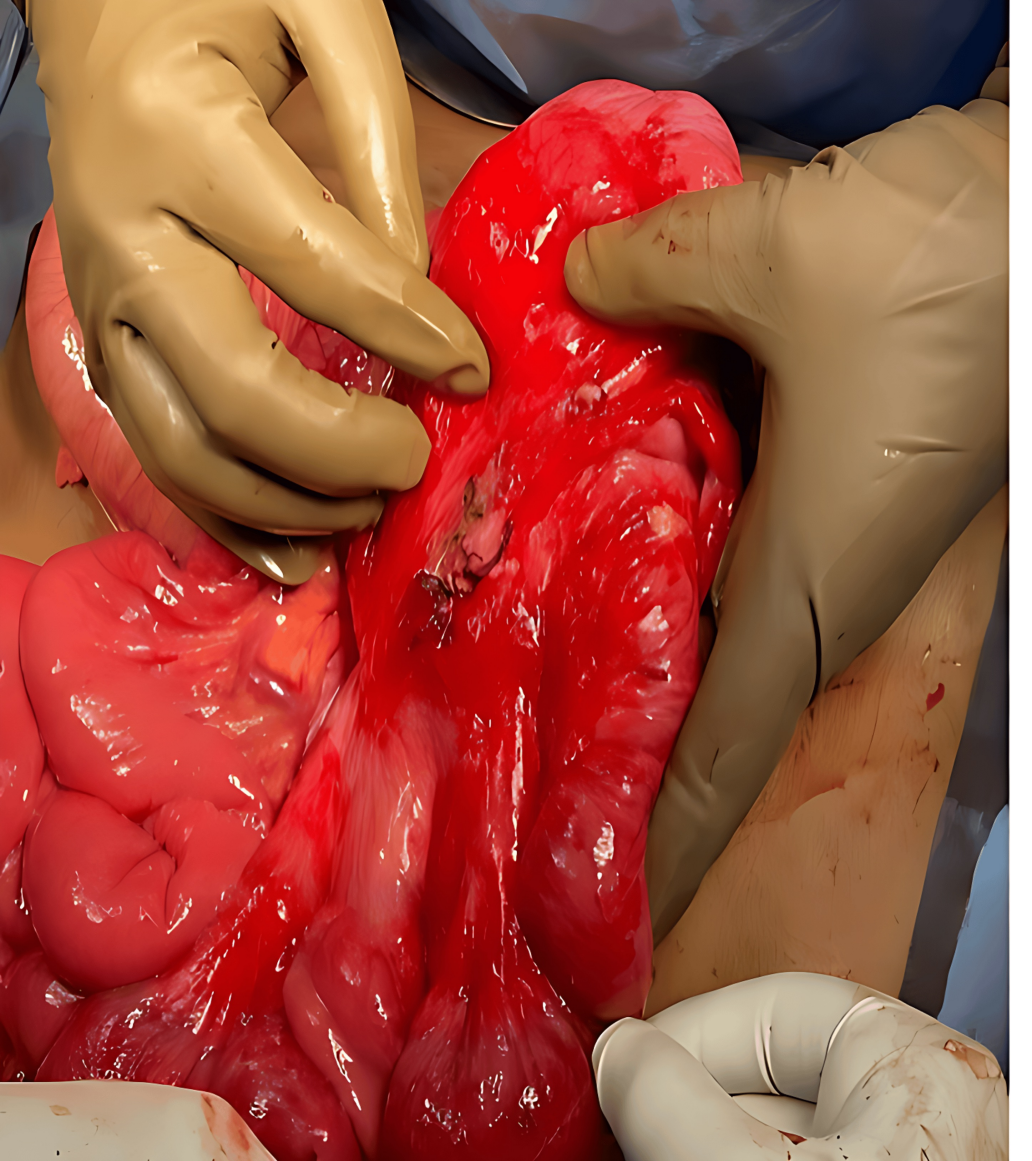
Intraoperative image

Based on the radiological findings of intestinal malrotation with midgut volvulus, the patient underwent emergency exploratory laparotomy. Intraoperatively, non-rotation of the midgut with volvulus and the cecum in the left pelvis was noted. A small mesenteric fold adhered to the retroperitoneum near the duodenojejunal flexure was identified. Congestion was observed in the terminal ileum, ileocecal junction, ascending colon, and cecum. The appendix appeared inflamed, and bilaterally fused kidneys were found near the midline, with an empty right renal fossa. The volvulus was untwisted, with the colon positioned in the left lateral gutter and the small bowel in the right lateral gutter (Figures 4-6).

The postoperative period was uneventful, and the patient was discharged on postoperative day 7.

## Discussion

Intestinal malrotation is most frequently identified during the initial month of life. However, it is crucial to recognize that the diagnosis can also be made in adults, indicating the potential for delayed presentation [2]. Given the frequently obscure clinical manifestations of intestinal malrotation in adults, the predominant symptoms reported by patients are vomiting and recurrent abdominal pain, likely arising from an underlying pattern of chronic intermittent bowel obstruction [6,7]. A subset of adult patients with intestinal malrotation may exhibit malabsorption secondary to both reduced oral intake and protein-losing enteropathy stemming from chronic or intermittent volvulus-induced diarrhea [1].

The diagnostic workup for suspected intestinal malrotation often involves several imaging modalities, including plain radiography, ultrasonography, and computed tomography. Nevertheless, contrast-enhanced studies of the stomach and duodenum have been shown to provide the most precise and dependable diagnostic information [8]. The "corkscrew sign," observed on imaging, is a hallmark of intestinal malrotation, arising from the uneven distension of various parts of the duodenum. Additionally, the abnormal location of the duodenojejunal junction, secondary to jejunal folding, represents another key radiological feature [9]. The superior mesenteric vein (SMV) being located in front of the SMA on ultrasound is one of the main signs of intestinal malrotation. Doppler ultrasound can show the whirlpool sign, which proves that the SMV is rotating around the SMA, a sign of pathology [10]. One of the ways computed tomography (CT) imaging is helpful is by locating the jejunal arteries on the right side of the superior mesenteric artery, which is not normal [11]. Considering that intestinal blockage due to malrotation is very common, most people with this condition are advised to have an elective laparotomy right away. Elective surgery for intestinal malrotation has usually been done using Ladd's procedure since 1936 [12]. The standard Ladd's procedure for elective intestinal malrotation entails detorsion of any midgut volvulus, division of obstructive peritoneal bands, non-rotation positioning of colon and small bowel, and prophylactic appendectomy. However, if there is bowel ischemia, removing the affected parts is crucial; therefore, Ladd's procedure cannot be used. Sometimes, resection of a large part of the small bowel can result in short bowel syndrome. Alternative treatments, including cecopexy, endoscopic reduction, and laparoscopic techniques, have been documented. Laparoscopic Ladd's procedure has shown promise for elective cases, potentially offering benefits over the traditional open approach [13]. However, surgical management of intestinal malrotation should be tailored to the individual patient.

This case highlights several key considerations regarding this infrequent condition. The clinical presentation of adult malrotation can be subtle, although the "whirlpool sign" on abdominal CT may suggest bowel torsion. Acute presentations, however, often involve extensive bowel necrosis, potentially requiring significant resection. The subsequent management of short bowel syndrome, a potential consequence of massive small bowel resection, is complex and frequently complicated by electrolyte abnormalities, malnutrition, immunodeficiency, organ failure, and sepsis. Refeeding enteroclysis can be a valuable tool among these patients, provided there is adequate residual healthy distal small bowel for nutrients, bile salt, and fluid absorption [14].

## Conclusions

Given the variable clinical presentation of intestinal malrotation, ranging from asymptomatic to vaguely symptomatic, this condition should be a key consideration in differential diagnosis of acute abdomen and intestinal ischemia. Accurate diagnosis, relying on the combination of history, physical examination, and imaging, is paramount to facilitate prompt surgical intervention aimed at limiting patient morbidity and mortality.
